# Restoring Hip Joint Anatomy With the Robotic Arm-Assisted System in Hip Fractures: Expanding the Applications for Hip Surgery

**DOI:** 10.1016/j.artd.2024.101381

**Published:** 2024-04-17

**Authors:** Konstantinos Dretakis, Maria Piagkou, Christos Koutserimpas

**Affiliations:** a 2^nd^ Department of Orthopaedics, “Hygeia” General Hospital of Athens, Athens, Greece; b Faculty of Health Sciences, Department of Anatomy, School of Medicine, National and Kapodistrian University of Athens, Athens, Greece

**Keywords:** Hip surgery, Robotics in orthopaedics, Hip joint anatomy, Hip prostheses, Hip fracture, Total hip arthroplasty

## Abstract

Total hip arthroplasty (THA) has improved the life quality of osteoarthritic patients, yet challenges persist. The robotic arm-assisted system, integrated into THA, aims to refine implant positioning, enhance precision, reduce errors, and restore the hip joint’s anatomy, including hip center, femoral offset, and limb length. We present the first use of the system for the treatment of a subcapital femoral neck hip fracture. A 62-year-old female suffering a left subcapital hip fracture underwent THA using the robotic arm-assisted system. After acetabular registration, accurate component placement was achieved, and a 9-mm limb length discrepancy was addressed. The patient had an unremarkable recovery with a reported Harris hip score of 96.5 at 18 months postsurgery. Robotic-guided navigation in THA, as showcased in this case, ensures accurate implant positioning by restoring the anatomical features of the hip joint. Its potential extends beyond conventional applications, hinting at future use in trauma, revision, and oncology cases. While promising, future adaptations should consider soft tissue dynamics to ensure joint stability and overall success.

## Introduction

Throughout time, arthroplasty methods and techniques have exhibited impressive progress [1,2]. The evolution of joint reconstruction surgery has embraced various techniques and technologies. Those include minimally invasive procedures, faster recovery strategies, improved preoperative, intraoperative, and postoperative care to reduce blood transfusions, as well as notable advancements in navigation and robotic systems [[3], [4], [5], [6]].

Total hip arthroplasty (THA) stands as the preferred surgical intervention for individuals in the advanced stages of osteoarthritis, markedly alleviating pain and enhancing hip functionality and overall quality of life [6]. While most patients experience favorable functional outcomes, there are issues to be addressed, such as component impingement, hip dislocation, discrepancies in leg length, increased wear on bearing surfaces causing periprosthetic bone resorption, and changes in hip biomechanics [1].

In recent times, there has been growing emphasis on incorporating robotics into THA with the expectation that it could open avenues for advancements [2,7]. Robotics’ integration could lead to more personalized implant positioning, enhanced precision, and reduction in surgical errors. Consequently, this is anticipated to yield improved patient outcomes, along with lower rates of revisions [7]. The robotic arm-assisted surgical system (MAKO Robotic Arm-assisted Total Hip, Stryker, Mako Surgical Corp., Fort Lauderdale, FL) enables personalized preoperative planning and facilitates robotic surgery utilizing 3-dimensional computed tomography (CT) models of the patient's hip, taking into account the patient’s anatomy, including acetabulum and femoral anteversion and size, hip joint’s offset, length discrepancy, as well as the possibility of anterior impingement [8]. This innovative technology aims to mitigate potential human errors and decrease intraoperative complication rates, as well as to increase implant survivorship [2,7].

Robotic arm-assisted THA has been used effectively for osteoarthritis cases [2,7]. THA has also been used for hip fractures of the femoral neck; however, the robotic arm-assisted system (RAAS) has not been utilized so far in these instances. Our objective is to recognize the potential advantages of robotic arm-assisted surgery in addressing femoral neck fractures and possibly expand its indications.

We present the first case of a subcapital femoral neck hip fracture treated with THA with the RAAS, expanding the possible applications of this system. We also discuss the future applications of this technology. Written informed consent has been obtained from the patient for the publication of this report.

## Case history

A 62-year-old female was transferred to the 2nd Orthopaedics Department of the “Hygeia” General Hospital of Athens, Greece, after sustaining a left subcapital femoral neck fracture 48 hours ago.

The patient was alert and hemodynamically stable (blood pressure: 143/77 mmHg, heart rate = 83 beats per minute, SpO2 = 99%). The patient had suffered a fall while walking and could not bear any weight on the injured lower limb. She did not sustain any other injuries, while her remaining medical history was unremarkable. Her left lower limb was shortened and in slight external rotation.

**Figure 1:**
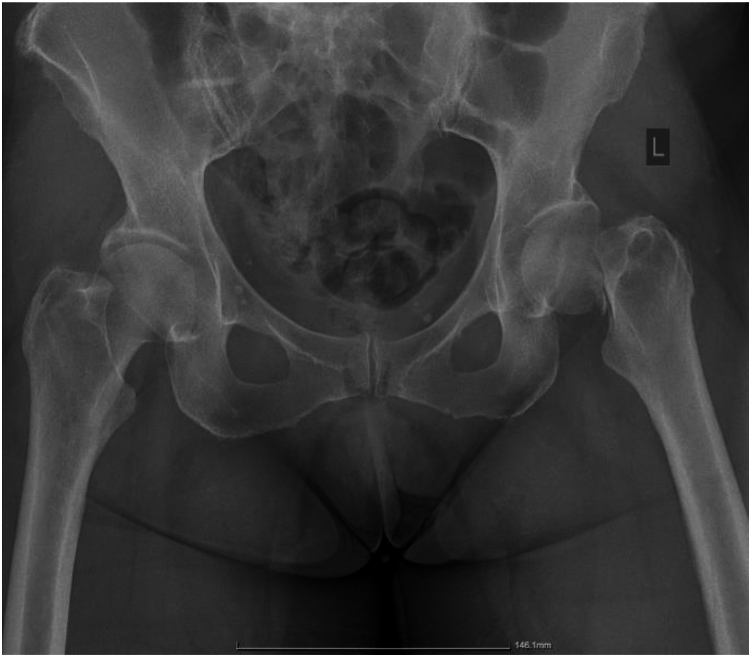
Anteroposterior X-ray of the pelvis, revealing a Garden III subcapital hip fracture.

The x-rays confirmed the diagnosis, and the fracture was classified as a Garden type III (Fig. 1). Following discussion with the patient about the possible surgical interventions, THA was decided to ensure better functional outcomes in this relatively young individual.

**Figure 2:**
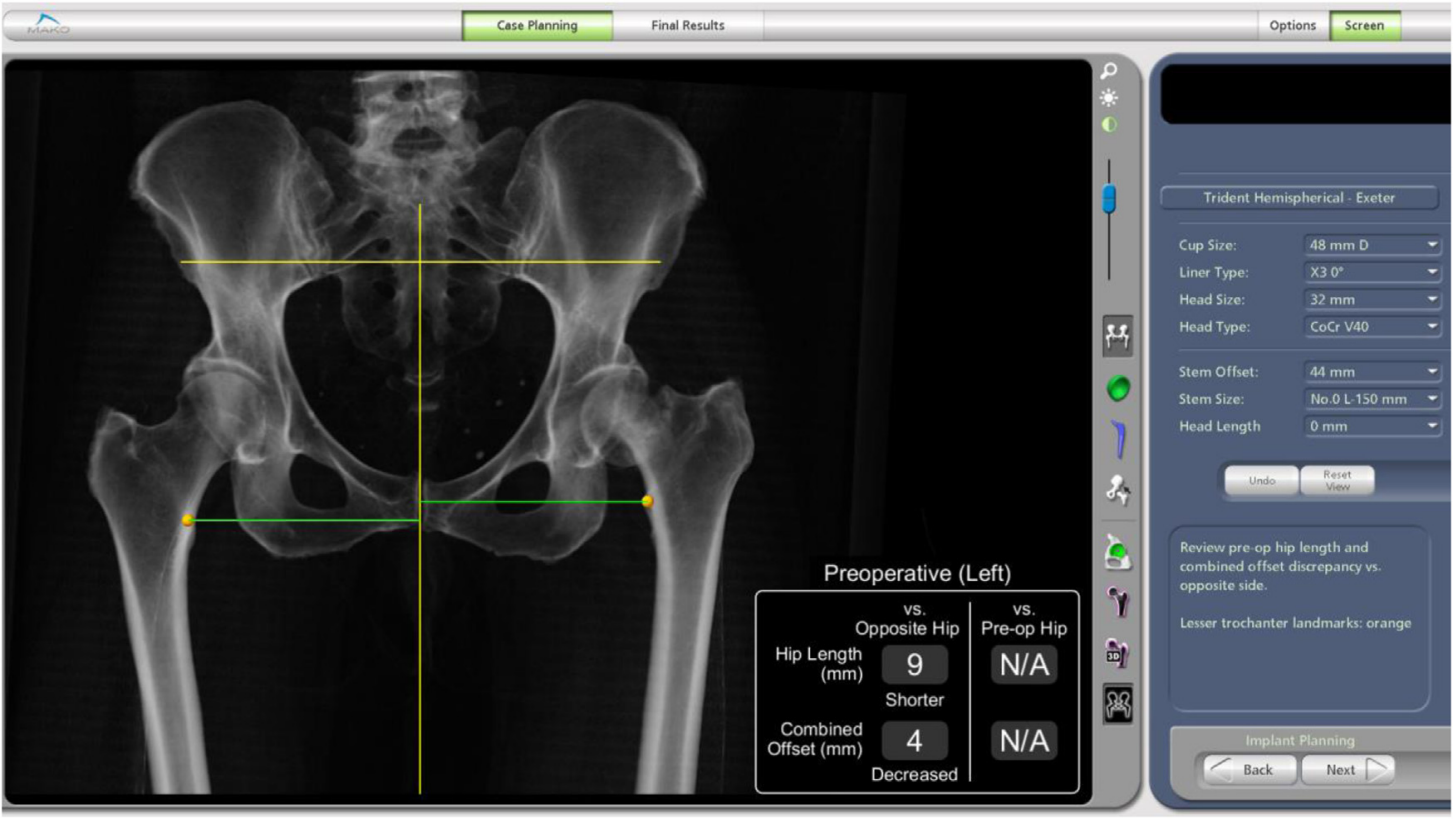
The surgical plan for the case, revealing the anatomical information of the patient.

**Figure 3:**
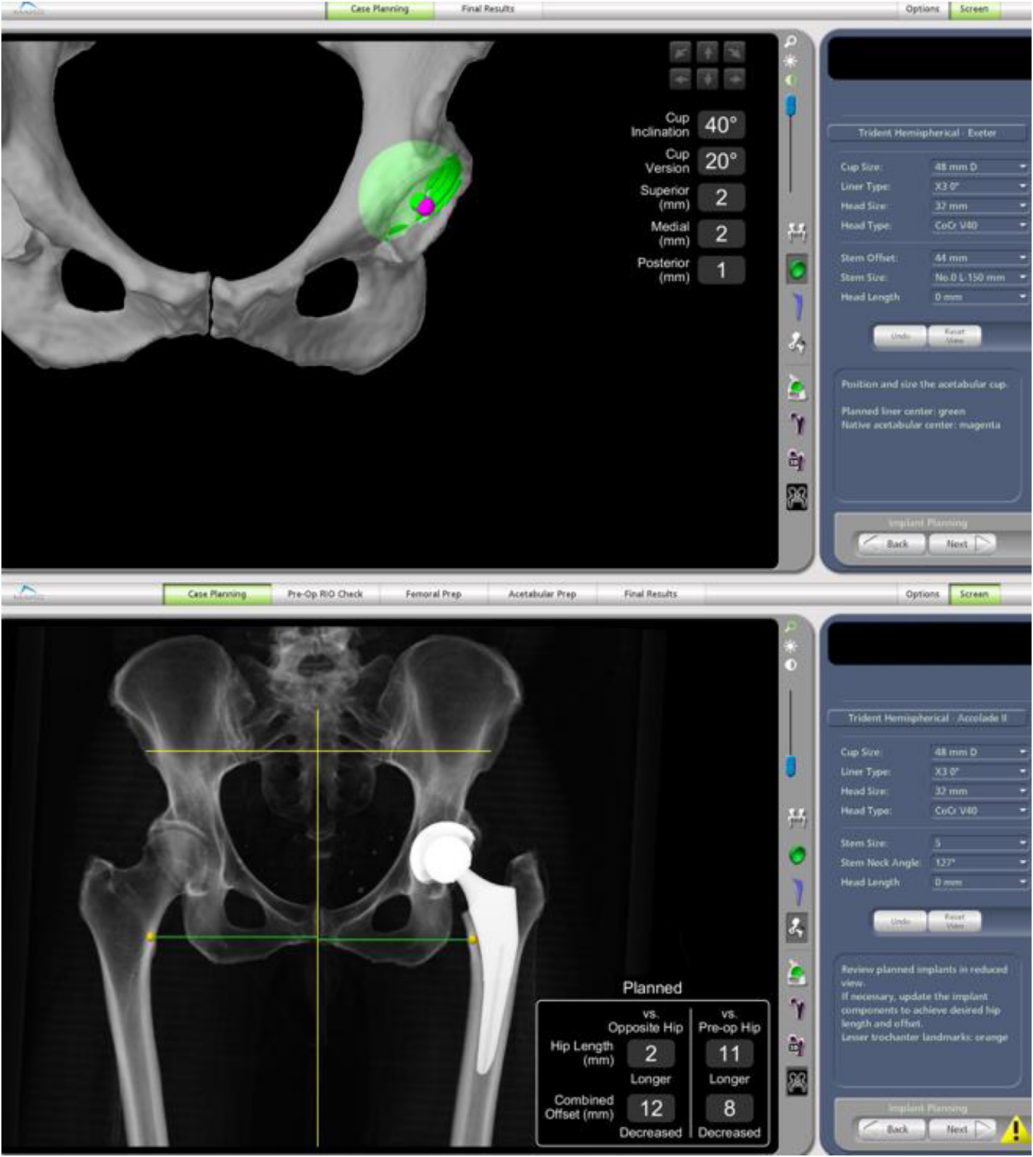
Planning of the prostheses positioning and size.

We decided to use the RAAS for this patient. Pelvic and bilateral knee CT scans were performed before the operation. These scans provided anatomical information that was then imported into the preoperative workstation for assessment, enabling virtual planning and execution. The RAAS utilizes this CT data to create personalized preoperative plans, aiding in selecting the right component sizes and ensuring precise placement of the stem and cup during surgery. Additionally, the 3-dimensional models generated from the pelvis and knee scans offer insights into native anatomy, encompassing pelvic tilt, leg length, and hip offset (Fig. 2). Preoperative planning revealed that the patient’s limb was 9 mm shorter due to the sustained fracture. After taking into account the patient’s specific anatomy, a size 5 femoral stem (Accolade II, Stryker, Kalamazoo, MI) was planned, and the acetabulum component (Trident Hemispherical cup, Stryker, Kalamazoo, MI) was set to 48 mm (Fig. 3). The CT scan and the planning were done within 12 hours after the patient’s transfer to the hospital. It should be noted that we had to expedite this procedure since this was a fracture patient.

Under epidural anesthesia, the patient was placed in a lateral position. The robotic arm was placed on the patient's right side, aligning it with the anterior superior iliac spine at the same level in order to achieve an angle of approximately 45° between the robotic arm and the operating table. Two minor incisions were made along the anterior edge of the opposite iliac crest, spaced approximately 1 cm apart. Two threaded pins are then placed into the thickest part of the iliac crest. The pelvic attachment device was affixed to them, facilitating a connection between the surgeon and the software via the infrared camera during the acetabular registration phase.

A modified Hardinge approach was used. The acetabulum and femoral checkpoints were also placed. After checking the limb length, the femoral head was removed, and an osteotomy of the remaining femoral neck was performed. The femoral cut was performed with the aid of the RAAS. In particular, the line of the cut is shown on the system’s monitor, according to planning. With the aid of the probe and taking into account 2 points (lesser trochanter and saddle point: the most distal part of the junction between the superior aspect of the femoral neck and the greater trochanter), we marked the cut.

Registration of the acetabulum was then performed, and a total of 32 points were placed into the cavity and all around the acetabulum edge were taken using the probe. Preparation of the acetabulum was performed with the robotic arm, and the cup was placed with the impactor. The position of the cup (48 mm Trident Hemispherical cup, Stryker, Kalamazoo, MI) was assessed using a probe to determine its inclination and anteversion (41° inclination and 19° anteversion). At that point, the femoral canal was prepared and the press-fit stem was placed (Accolade II no 5, Stryker, Kalamazoo, MI). Trial reduction with a standard 22.2 mm head was performed showing good stability and leg length. The modular dual mobility system was placed including the 38mm CoCr liner (external bearing) and the 38D X3 polyethylene insert (Stryker, Kalamazoo, MI). The 22.2 mm standard V40 femoral head was then placed. Final measurements revealed that the injured limb was 2mm longer than the opposite healthy one.

**Figure 4:**
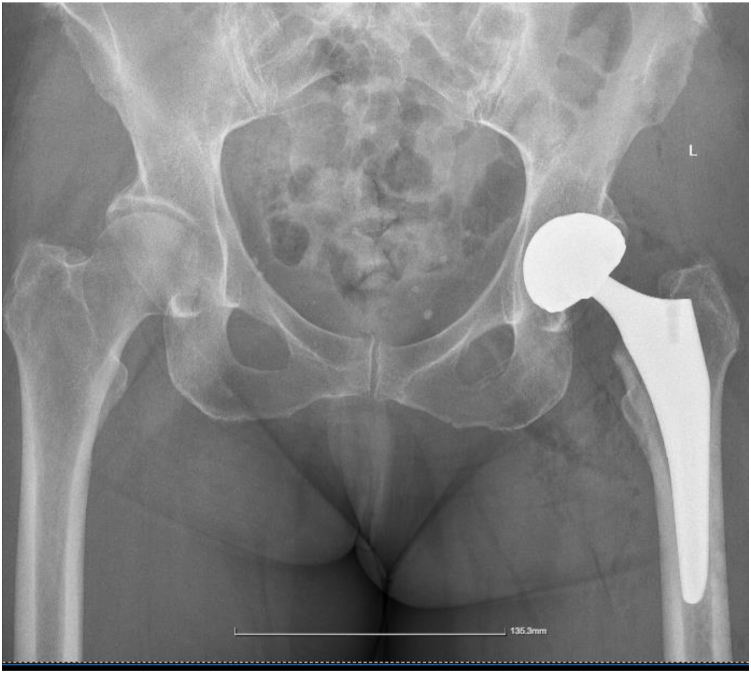
Postoperative anteroposterior X-ray view of the pelvis.

The patient had an uneventful recovery (Fig. 4). She was discharged on the third postoperative day. During the first 4 months, she had a slight Trendelenburg gait, which ameliorated. Eighteen months after the surgery, she has no restrictions in her everyday life, continues walking, and works as a teacher. Moreover, she has a reported Harris hip score of 96.5.

## Discussion

Ensuring a biomechanically balanced hip prosthesis aims to reduce strain on the implanted components, enhancing overall stability and durability [1,7]. The RAAS aims to increase the accuracy of the surgical plan’s execution [8,9]. Misalignment of components has been associated with increased occurrences of hip dislocations, compromised biomechanics, increased wear rates, discrepancies in leg length, and the necessity for revision surgeries [9,10]. The RAAS evaluates the patient’s specific anatomy in order to achieve a personalized surgical plan and offers accuracy in the execution [10,11]. This is the main reason that we chose to use the system in this case, since the patient was relatively young with an active lifestyle. The present case represents the first report of the use of this system for the treatment of a subcapital femoral neck fracture. Additionally, we used dual mobility THA since this was a fracture case that is more prone to dislocations, while a CoCr liner was placed since it is a durable, biocompatible implant that can withstand the mechanical stresses of daily activities and contribute to long-term joint function and stability [12]. Typically, hip fracture patients exhibit excellent range of motion before surgery and rarely experience significant contractures [13]. However, this, combined with the potential for postoperative confusion, which is common among elderly hip fracture cases, contributes to an increased risk of dislocation. When compared to the standard THA, the design of the dual mobility THA enhances the effective head size and head-to-neck ratio. This enhancement subsequently augments the range of motion and reduces the risk of dislocation [13]. This is the main reason that we used dual mobility in the reported case.

To establish a tailored plan, it is crucial to identify specific femoral and acetabular morphological characteristics [[14], [15], [16]]. During the preoperative phase, the robotic software offers various data points, including the final size of prosthetic components, skeletal orientation, and the inclination and version of the acetabular cup and femoral stem [14]. These details require comparison with the opposite hip to address any anatomical differences [9,14,15]. In this patient, we were able to measure the leg length discrepancy and assess the inclination and anteversion of the acetabulum, as shown in Figure 3. The sizing of both the acetabulum cup and the femoral stem were also decided during planning.

It should be noted that there are some limitations to the system as well. One significant obstacle of the RAAS is its considerable expense, which encompasses not just the initial capital outlay but also the ongoing costs associated with maintenance and the need for disposable materials and imaging resources [7]. Additionally, the preoperative CT imaging required imposes a burden of radiation exposure on the patient, estimated to equate to approximately 48 chest X-rays in dosage, as well as a delay in surgery, which also represents a major concern for hip fracture patients [17]. As stated, we had to expedite the procedure of surgical planning, minimizing this timeframe to less than 12 hours. Nevertheless, this does not reflect common practice. Finally, the pins represent potential infection sites as well as causes of pelvic rim fractures, especially in osteoporotic patients [7,18].

Although the indication of this system for THA is hip osteoarthritis, we believe we have demonstrated that it may prove very useful in femoral neck fracture cases that require THA. It is of note that the RAAS does not take into consideration the soft tissues; hence, the stability of the joint should always be checked manually, and further changes in the preoperative plan should be performed accordingly.

The use of robotic-arm-assisted surgery holds promise for enhancing hip joint biomechanics, presenting a valuable innovation in THA. Future applications may include traumatology as well as oncological cases. Furthermore, it should be noted that the system could also expand to other joints, such as the shoulder and ankle, while also being used for revision cases.

## Summary

Robotic-guided navigation stands as a promising solution to enhance outcomes, offering consistent and foreseeable results. Through robotic navigation and the utilization of personalized preoperative planning, it becomes feasible to strategize the appropriate combined anteversion of the stem and cup, establish a precise hip center of rotation, and reinstate the natural offset and leg length. This aspect is crucial in preventing complications and early mechanical issues such as hip impingement, dislocation, or muscle imbalances. The robotic arm-assisted technology may be used with adaptations in trauma cases, while in the near future it could include revision and oncology cases.

## Acknowledgments

The publication of the article in OA mode was financially supported in part by HEAL-Link.

## Conflicts of interest

K. Dretakis is a paid instructor for Stryker. All other authors declare no potential conflicts of interest.

For full disclosure statements refer to https://doi.org/10.1016/j.artd.2024.101381.

## Informed patient consent

The author(s) confirm that written informed consent has been obtained from the involved patient(s) or if appropriate from the parent, guardian, power of attorney of the involved patient(s); and, they have given approval for this information to be published in this case report (series).

## CRediT authorship contribution statement

**Konstantinos Dretakis:** Writing – review & editing, Supervision, Project administration, Methodology, Investigation, Data curation, Conceptualization. **Maria Piagkou:** Writing – review & editing, Validation, Supervision, Investigation, Formal analysis, Conceptualization. **Christos Koutserimpas:** Writing – review & editing, Writing – original draft, Methodology, Investigation, Conceptualization.

## Appendix A Supplementary Data

Conflict of Interest Statement for Dretakis

Conflict of Interest Statement for Koutserimpas

Conflict of Interest Statement for Piagkou

## References

[1] Perazzini P., Trevisan M., Sembenini P., Alberton F., Laterza M., Marangon A. (2020). The Mako robotic arm-assisted total hip arthroplasty using direct anterior approach: surgical technique, skills and pitfals. Acta Biomed.

[2] Ando W., Takao M., Hamada H., Uemura K., Sugano N. (2021). Comparison of the accuracy of the cup position and orientation in total hip arthroplasty for osteoarthritis secondary to developmental dysplasia of the hip between the Mako robotic arm-assisted system and computed tomography-based navigation. Int Orthop.

[3] Besiris G.T., Koutserimpas C., Karamitros A., Karaiskos I., Tsakalou D., Raptis K. (2020). Topical use of tranexamic acid in primary total knee arthroplasty: a comparative study. G Chir.

[4] Kalavrytinos D., Koutserimpas C., Kalavrytinos I., Dretakis K. (2020). Expanding robotic arm-assisted knee surgery: the first attempt to use the system for knee revision arthroplasty. Case Rep Orthop.

[5] Koutserimpas C., Piagkou M., Karaiskos I., Karamitros A., Raptis K., Kourelis K. (2023). Modified anterolateral minimally invasive surgery (ALMIS) for total hip replacement: anatomical considerations, range of motion and clinical outcomes. Medicina (Kaunas).

[6] Learmonth I.D., Young C., Rorabeck C. (2007). The operation of the century: total hip replacement. Lancet.

[7] Bullock E.K.C., Brown M.J., Clark G., Plant J.G.A., Blakeney W.G. (2022). Robotics in total hip arthroplasty: current concepts. J Clin Med.

[8] Marchand R., Olsen D., Shul C., Edmond T., Hameed D., Angerett N. (2023). Mako robotic-arm assisted total hip arthroplasty: avoiding impingement with updated THA software. Surg Technol Int.

[9] Sato K., Sato A., Okuda N., Masaaki M., Koga H. (2023). A propensity score-matched comparison between Mako robotic arm-assisted system and conventional technique in total hip arthroplasty for patients with osteoarthritis secondary to developmental dysplasia of the hip. Arch Orthop Trauma Surg.

[10] Kim K., Kwon S., Kwon J., Hwang J. (2023). A review of robotic-assisted total hip arthroplasty. Biomed Eng Lett.

[11] Subramanian P., Wainwright T.W., Bahadori S., Middleton R.G. (2019). A review of the evolution of robotic-assisted total hip arthroplasty. Hip Int.

[12] Hu C.Y., Yoon T.R. (2018). Recent updates for biomaterials used in total hip arthroplasty. Biomater Res.

[13] Ma H.H., Chou T.A., Pai F.Y., Tsai S.W., Chen C.F., Wu P.K. (2021). Outcomes of dual-mobility total hip arthroplasty versus bipolar hemiarthroplasty for patients with femoral neck fractures: a systematic review and meta-analysis. J Orthop Surg Res.

[14] Tarwala R., Dorr L.D. (2011). Robotic assisted total hip arthroplasty using the MAKO platform. Curr Rev Musculoskelet Med.

[15] St Mart J.P., Goh E.L., Shah Z. (2020). Robotics in total hip arthroplasty: a review of the evolution, application and evidence base. EFORT Open Rev.

[16] Guo D.H., Li X.M., Ma S.Q., Zhao Y.C., Qi C., Xue Y. (2022). Total hip arthroplasty with robotic arm assistance for precise cup positioning: a case-control study. Orthop Surg.

[17] Dretakis K., Koutserimpas C. (2024). Pitfalls with the MAKO robotic-arm-assisted total knee arthroplasty. Medicina (Kaunas).

[18] Bagaria V., Sadigale O.S., Pawar P.P., Bashyal R.K., Achalare A., Poduval M. (2020). Robotic-assisted knee arthroplasty (RAKA): the technique, the technology and the Transition. Indian J Orthop.

