# Supplementary material for: Restoring Hip Joint Anatomy With the Robotic Arm-Assisted System in Hip Fractures: Expanding the Applications for Hip Surgery
Source: Arthroplast Today. 2024 Apr 17;27:101381. doi: 10.1016/j.artd.2024.101381 (PMC11047285; doi:10.1016/j.artd.2024.101381)
Supplement: Conflict of Interest Statement for Dretakis [file mmc1.pdf]

CONFLICT OF INTEREST STATEMENT

American Association of Hip and Knee Surgeons

(Adopted from the American Academy of Orthopaedic Surgeons disclosure statement)

The following form **must be filled out completely and submitted by each author (example, 6 authors, 6 forms).**
All items require a response. If there is no relevant disclosure for a given item, enter "*None*."

Manuscript Title: **Restoring hip joint anatomy and biomechanics with the robotic arm-assisted system (MAKO) in hip fractures: Expanding the applications for hip surgery**

---

1. Royalties from a company or supplier (The following conflicts were disclosed)

None

2. Speakers bureau/paid presentations for a company or supplier (The following conflicts were disclosed)

None

3A. Paid employee for a company or supplier (The following conflicts were disclosed)
None

3B. Paid consultant for a company or supplier (The following conflicts were disclosed)
KD is a paid instructor for Stryker.

3C. Unpaid consultants for a company or supplier (The following conflicts were disclosed)

None

4. Stock or stock options in a company or supplier (The following conflicts were disclosed)

None

5. Research support from a company or supplier as a Principal Investigator (The following conflicts were disclosed)

None

6. Other financial or material support from a company or supplier (The following conflicts were disclosed)

None

7. Royalties, financial or material support from publishers (The following conflicts were disclosed)

None

8. Medical/Orthopaedic publications editorial/governing board (The following conflicts were disclosed)

None

9. Board member/committee appointments for a society (The following conflicts were disclosed)

Each author must sign AND print or type his/her name, date and submit a separate form

In addition, one BLINDED Conflict of Interest form (no author names used) should be submitted per manuscript with all author disclosures.

K Dretakis

Author Name (Print or Type)

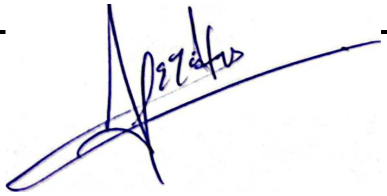

26/12/2023

Date
